# Chemical Investigation on the Volatile Part of the CO_2_ Supercritical Fluid Extract of Infected *Aquilaria sinensis* (Chinese Agarwood)

**DOI:** 10.3390/molecules29102297

**Published:** 2024-05-14

**Authors:** Marko Z. Mladenović, Ou Huang, Bo Wang, Alexandre Ginestet, Didier Desbiaux, Nicolas Baldovini

**Affiliations:** 1 Faculty of Sciences and Mathematics, University of Niš, Višegradska 33, 18000 Niš, Serbia; markohem87@gmail.com; 2 Guangdong Shangzhengtang Group Co., Ltd., 6 Qian Wu Street, Dongcheng Road, Dongguan 523129, China; sztchina@163.com; 3 School of Life and Health Technology, Dongguan University of Technology, Dongguan 523808, China; bwang@dgut.edu.cn; 4 MAG Industry Holdings Ltd., 26 Queensland, Nancheng Century City, Dongguan 523617, China; alexandre@mag-vision.fr (A.G.); didier.desbiaux@gmail.com (D.D.); 5 Institut de Chimie de Nice, UMR 7272, Université Côte d’Azur, Parc Valrose, 06108 Nice, France

**Keywords:** agarwood, *Aquilaria sinensis*, supercritical fluid extraction, eremophilane sesquiterpenoids, neopetasane

## Abstract

This work is focused on the characterization of the composition of a CO_2_ supercritical fluid extract of *Aquilaria sinensis* (Chinese agarwood) collected in the Dongguan area (China) and infected by mechanical methods. The constituents of this extract were analyzed by gas chromatography–mass spectrometry (GC-MS) and quantified accurately by gas chromatography with a flame ionization detector (GC-FID), using an internal reference and predicted response factors. Since a significant number of components of this extract remained non-identified after the initial GC-MS analysis of the whole extract, its fractionation by chromatography on silica gel helped to characterize several additional constituents by isolation and structural analysis by NMR spectroscopy. The main components are the classical agarwood chromones (Flindersia chromone and its mono-, di-, and trimethoxylated analogues (respectively, 11.01% and 0.11–4.02%) along with sesquiterpenic constituents typically found in agarwood essential oils, like baimuxinal (1.90%) and kusunol (1.24%), as well as less common selinane dialdehydes (1.58–2.27%) recently described in the literature. Moreover, the structure and stereochemistry of a new sesquiterpenic alcohol, 14β,15β-dimethyl-7αH-eremophila-9,11-dien-8β-ol (0.67%), was determined unambiguously by the combination of structural analysis (NMR, MS), hemisynthesis, and total synthesis, leading to dihydrokaranone and a neopetasane epimer.

## 1. Introduction

Agarwood is a highly valuable resinous wood produced by various tree species, mostly from the genera *Aquilaria* and *Gyrinops* (Thymelaeaceae) endemic to the south east of Asia. The wood of healthy agarwood-producing species is generally light, pale yellow, devoid of resin, and of little value. However, when the trees are infected by fungi and microorganisms, their wood turns to a dense and dark resinous material which constitutes agarwood, the most expensive wood in the world. In East Asia, agarwood is also known as c’hen siang (chinese), trầm hương (vietnamese), and jinkô (japanese), all of these names meaning “sinking incense wood” since the most infected pieces are often denser than water, and are traditionally burned as incense for perfuming purposes. In addition to its use as a highly appreciated fragrance, agarwood is an important component in traditional Chinese and Ayurvedic medicines for a variety of applications [1,2], and its complex chemical composition [3,4] combined with its prestigious status among aromatic natural raw materials still continues to fascinate fragrance chemists. Because of its high economic value, agarwood soon became one of the main natural materials traded between Asian nations along the Maritime Silk Road [5,6]. Nowadays, the biggest consumer of agarwood is the Middle East, where it has been used for more than 2000 years [7] as highly precious incense for daily life and religious ceremonies. Since no *Aquilaria* species are endemic in this area, a significant part of the agarwood consumed in the Middle East is produced from *A. malaccensis* and *A. crassna* cultivated in the main producing countries (Indonesia, Malaysia, Thailand, Vietnam, etc.). In contrast, the agarwood obtained from the endemic species in China, *A. sinensis,* represents only a small part of the global agarwood market, despite its use in China since at least the sixth century. Indeed, it is likely that the utilization of agarwood began during the Tang Dynasty in the southern provinces of China (Guangdong, Guangxi, Hainan Island). However, some records of the historical events that occurred during the Sui Dynasty (581–618), when Emperor Sui Yangdi burned large quantities of agarwood on a hill “to embalm all the country” [8], indicate that its use is even more ancient. 

For perfuming purposes, agarwood is traditionally burned as such, but alternatively it can also be hydrodistilled to produce an essential oil, which became a prestigious ingredient for fine perfume formulations. Compared to agarwood essential oils, SFE extracts are much less common, and their composition has received less attention in the scientific literature, even if some authors reported the analysis of SFE extracts of *A. crassna* [9,10]. However, since SFE extraction is a more environment-friendly process for agarwood valorization, it may represent a promising alternative for the production of agarwood-derived raw materials. Therefore, there is a strong need to better characterize the composition of these extracts before considering any commercial development. Moreover, from the phytochemical point of view, SFE extraction has a lower tendency to generate thermal and chemical artifacts compared to hydrodistillation, so the characterization of the composition of SFE extracts is a useful approach to identify genuine constituents of the starting plant material. In the frame of our investigations on the phytochemistry of *Aquilaria sinensis*, we collected agarwood in one of the four former royal incense gardens, within the core production zone of Dongguan agarwood, and performed its CO_2_ supercritical fluid extraction (SFE). In the present article, we wish to report the results of our analysis of the SFE extract of Dongguan agarwood using a combination of GC-MS, fractionations followed by structural analyses, and the hemisynthesis and total synthesis of several eremophilane derivatives to determine unambiguously the structure of one of the constituents of the extract.

## 2. Results and Discussion

The constituents identified in the SFE extract are reported in Table 1, with their mass percentages determined by GC-FID using predicted response factors [11]. A total of 34 constituents representing 36.0% of the analyzed sample were identified by comparison of their retention indices and mass spectra with those contained either in the literature or in our laboratory-made database built with authentic substances. In addition, more than 91 compounds were detected in the GC chromatogram but remained non-identified. By attributing a mean response factor of 0.80 (using tetradecane as an internal reference) to these unknown compounds, the total mass percentage of identified and non-identified constituents reaches only 54.9% of the entire mass, a low amount that is certainly due in part to the presence of poorly volatile compounds not eluted in the GC column, as can be expected for SFE extracts.

**Figure 1:**
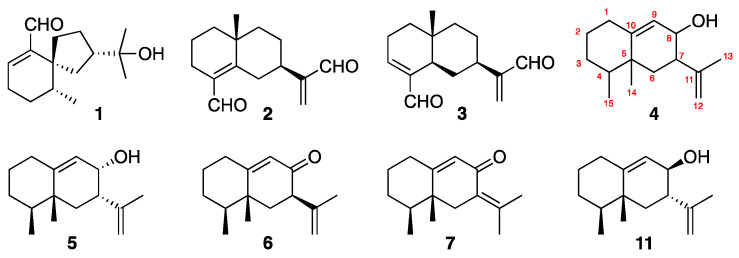
Structures of some sesquiterpenic constituents of *A. sinensis* SFE extract (**1**–**4** and **6**–**7**) and related eremophilane sesquiterpenoids (**5**, **11**). Numbering of carbons in red on structure 4.

Not surprisingly, the most abundant constituent was Flindersia chromone (2-(2-phenylethyl)chromone) [13], followed by its mono-, di-, and trimethoxylated analogues. The exact position of the methoxy substituents on these chromones could not be established with certainty, due to the lack of reference compounds and the generally similar mass spectra of isomers. Moreover, among the unknown compounds mentioned above, many additional constituents produced MS spectra evoking compounds of the chromone family. As expected, the other constituents were mainly sesquiterpenic alcohols classically found in agarwood essential oils. The spirovetivane baimuxinal (oxoagarospirol) **1** [14,15] (Figure 1) was identified among the most abundant sesquiterpenoids, and in our experience, this compound is generally present in higher amounts in the essential oils produced from *A. sinensis* than in the oils from other species. Two constituents eluting after **1** (at RI = 1896 and 1924) were even more abundant, and their MS spectra evoked dioxygenated sesquiterpenoids. To characterize these compounds, we fractionated the extract by several successive column chromatographies on silica gel and silica gel containing 10% silver nitrate. Detailed NMR analyses of the pure samples helped us to characterize these two compounds as the known selinane dialdehydes (**2** and **3**) reported first by Bohlmann as constituents of *Libanothamnus occultus* [16]. The presence of these compounds in agarwood is known, as **2** could also be identified in *A. malaccensis* [17], and both **2** and **3** were detected later in *A. sinensis* [18].

With further chromatographic separations of the fractions obtained when trying to isolate **2** and **3**, we could also obtain another compound not identified in the first GC-MS screening. This constituent, eluting at RI = 1640, displayed an NMR spectrum showing the characteristic signals of a secondary allylic alcohol and an isopropenyl moiety. A detailed analysis of the gradient homonuclear ^1^H–^1^H correlation spectroscopy (COSY), heteronuclear single quantum coherence (HSQC), and heteronuclear multiple-bond correlation spectroscopy (HMBC) interaction (Table 2, and Supplementary Information) correlations led us to conclude that it was an eremophilane alcohol of general structure **4**. 

**Scheme 1:**
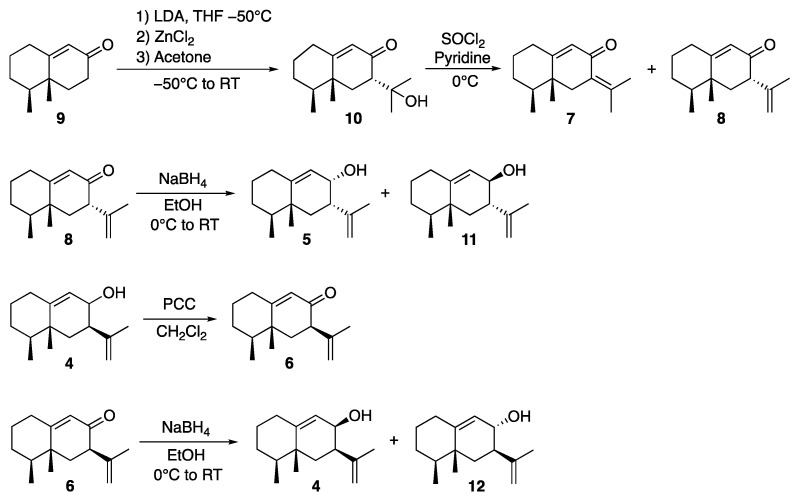
Syntheses and hemisyntheses of eremophilanes.

So far, the only isomer of **4** reported as a natural product is 14β,15β-dimethyl-7βH-eremophila-9,11-dien-8α-ol **5**, isolated from the liverworts *Marsupella emurginata* and *M. aquatica* [19,20]. However, since the published NMR data of **5** were slightly different from those of our isolated compound **4**, we suspected that it could be another stereoisomer. At first sight, **4** is formally a reduced analogue of neopetasane **6**, another classical component of agarwood [21], which easily isomerizes to dihydrokaranone **7**. Both **6** and **7** were also identified in our extract and are highly important odorant constituents of agarwood [21]. Indeed, **7** has been considered as a key contributor to the characteristic fragrance of agarwood [15], and several synthetic and hemisynthetic strategies to prepare **7** have been published and patented [22,23,24,25,26,27,28]. The synthesis of Hagiwara [24] caught our attention since it describes the preparation of **7** via the 7βH dihydrokaranone epimer **8**, which would be a useful precursor for structure confirmation in the context of our study. Hence, we synthesized **8** by first preparing octalone **9** via Wieland–Miescher ketone [29], and, following Hagiwara’s procedure [24], we added the enolate of **9** on acetone in the presence of zinc chloride to produce alcohol **10**, which was subsequently dehydrated with thionyl chloride in pyridine to a mixture of **7** and **8** (Scheme 1). This mixture could be fractionated by CC on SiO_2_-AgNO_3_ to deliver a pure sample of **8**. 

In his study, Hagiwara demonstrated the 7βH configuration of **8** by showing that this compound was not identical to a natural sample of the previously described **6** [24], identified at first in *Petasites hybridus* [30]. Moreover, **6** was later isolated from agarwood by Ishihara et al. [21] who confirmed unambiguously its absolute and relative stereochemistry by synthetic transformations. In our study, the comparison of the chromatographic and spectral data of synthetic **8** with those of **6** isolated from our extract confirmed that both compounds were different. Then, we reduced our synthetic sample of ketone **8** by sodium borohydride and obtained a mixture of two isomeric alcohols, which were fractionated by CC on silica gel to provide a pure sample of the main isomer **11** and a mixture of **11** with the minor isomer. The careful NMR analysis of **11** and the examination of the ^13^C NMR spectrum of the mixture led us to conclude that **11** was the 14β,15β-dimethyl-7βH-8β-hydroxy- isomer while its accompanying epimer was logically the known compound **5** cited above. Since the NMR data of **11** did not coincide with those of our isolated alcohol **4**, we deduced that this latter compound displayed a 7αH configuration, and this hypothesis was corroborated when we oxidized a sample of **4** with pyridinium chlorochromate and obtained a single product showing the same RI and NMR data as **6**.

**Figure 2:**
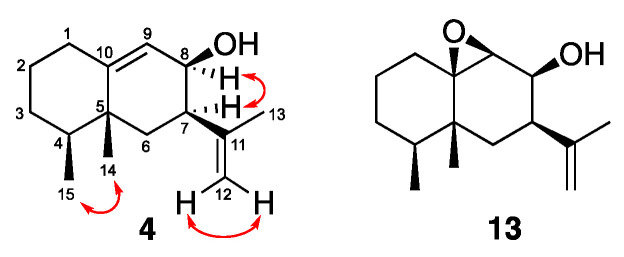
Structure of **4** with number of carbon atoms, stereochemistry, and key NOESY interactions; and structure of a known related agarwood constituent **13**.

Furthermore, the sodium borohydride reduction of the hemisynthetic sample of **6** produced a mixture in which alcohol **4** could be detected by GC-MS and ^1^H NMR, together with its suspected epimer **12**. Finally, the 8β-hydroxy configuration of **4** was established by considering the NOESY correlations between H7 and H8 (Figure 2). 

In conclusion, compound **4** is 14β,15β-dimethyl-7αH-eremophila-9,11-dien-8β-ol, and this stereochemistry is finally not surprising if we consider its plausible biosynthetic link with **6** or with its epoxy analogue **13** already identified in *A. malaccensis* [17]. The comparison of the chemical composition of this SFE extract with that of agarwood essential oils obtained from *A. sinensis* or other *Aquilaria* species clearly demonstrates that its content of chromones is much higher. This result can be easily explained since the poor volatility of these compounds significantly limits their extractability by the hydrodistillation procedure. Therefore, agarwood SFE extracts may represent promising materials for medicinal applications based on the pharmacological properties of the chromones, as these metabolites often display various interesting activities [4]. However, in view of the high number of non-identified constituents contained in these extracts, further phytochemical investigations are required to fully understand their richness and their potential.

## 3. Materials and Methods

### 3.1. General Procedures

Chemicals for synthesis were purchased from Sigma–Aldrich (L’Isle d’Abeau, France), Macherey-Nagel (Hoerdt, France), and VWR International (Fontenay-sous-Bois, France). All solvents (tetrahydrofuran (THF), diethyl ether (Et_2_O), dichloromethane (DCM), petroleum ether, and methyl tertbutyl ether (MTBE) were used as received, except THF which was purified by distillation on sodium/benzophenone before use. NMR analyses were performed on Bruker NanoBay Avance IIIHD (400 MHz) spectrometer and Bruker Avance DRX 500 (500 MHz) spectrometers (Bruker, FR-Wissembourg) at 25 °C in CDCl_3_ and C_6_D_6_. 

### 3.2. Plant Material and Extraction Procedure

The agarwood samples were collected in Dongguan’s agarwood intangible cultural heritage conservation garden, an area of 3400 acres located in Baihuadong, Dalingshan County, Dongguan City, China (latitude: 22°45′43″ N, longitude: 113°48′45″ E). The infected part of the *Aquilaria sinensis* wood (i.e., agarwood), displaying the characteristic brown discoloration, was carefully separated from the yellow-white healthy part, and processed as follows: Powdered agarwood (8000 g) was extracted by supercritical fluid extraction (SFE) using an HA220-40-11-C supercritical CO_2_ extractor (Nantong Huaan Supercritical Extraction Co., Ltd.; Nantong, China) at a temperature of 60–65 °C for 2.5 h. The extraction pressure was 29 Mpa, and the CO_2_ flow was 255 m^3^/h. The extraction yield was 0.12%, a higher yield than the one obtained by classical hydrodistillation of the material, as expected from this efficient extraction technique [31].

### 3.3. GC-MS Analyses

Gas chromatography–mass spectrometry (GC-MS) analyses were carried out using an Agilent 6890N gas chromatograph coupled to an Agilent 5973N mass-selective detector working in electron impact (EI) mode at 70 eV (scanning over 40–450 amu range in SCAN mode). The gas chromatograph was equipped with a fused silica capillary column, RTX-1 (30 m × 0.25 mm i.d., film thickness: 0.25 µm, Restek). The analytical parameters were the following: The carrier gas was helium at a flow rate of 1 mL/min, and the oven temperature was programmed from 60 to 250 °C at 3 °C/min and held isothermal for 20 min. Samples (1 μL of a ca. 10% solution of the isolated/synthesized compounds in MTBE) were injected in split mode (ratio: 1/100), and the injector temperature was 250 °C. The temperatures of the ion source and transfer line were 230 and 280 °C, respectively. LRI linear retention indices (RIs) were determined from the retention times of a series of *n*-alkanes with linear interpolation. The SFE extract or SFE extract chromatographic fraction samples were diluted (to a 5–10% concentration) in methyltertbutylether (MTBE) before injection. The constituents were identified by comparison of their mass spectra and LRI with those of pure compounds registered in commercial libraries and the literature data, and with a laboratory-made database built from authentic compounds. The identification and the FID quantitation of the constituents were made with the help of SearchReview (version 127) software (N. Waleson, Leoson, 2021).

### 3.4. GC-FID Analyses

Gas chromatography–flame ionization detector (FID) analyses were performed in the same chromatographic conditions as those described for GC-MS analyses, but with the following parameters concerning the detector: FID temperature set at 270 °C, and hydrogen and air flows set at 40 and 450 mL/min, respectively. The SFE extract samples were diluted (ca.1/10) in MTBE, spiked with an accurately weighted amount of internal standard for quantification (1% solution of tetradecane in toluene), and 1 μL of these solutions was injected in split mode (ratio 1/50). The quantification of the constituents was performed using predicted response factors, as described by Tissot et al. [11].

### 3.5. SFE Extract Fractionation

The SFE extract (2.025 g) was fractionated by several successive column chromatographies on silica gel and silica gel containing 10% silver nitrate (40–60 μm and 15–40 μm), using a gradient of petroleum ether and MTBE to isolate the following constituents: **2** (19 mg), **3** (10 mg), and **4** (8 mg).

12,15-dioxo-selinan-4,11-diene (**2**): colorless oil; ^1^H NMR data consistent with the literature [16]; ^13^C NMR data: ^13^C NMR (101 MHz, CDCl_3_) δ 194.26, 190.87, 162.66, 153.63, 133.40, 133.30, 41.38, 39.48, 38.52, 36.70, 29.06, 26.82, 25.23, 23.89, 17.87. EI-MS (70 eV) *m*/*z* (%): 232 (93, M^•+^), 217 (47), 214 (72), 203 (45), 199 (52), 175 (52), 171 (39), 147 (39), 137 (41), 105 (50), 91 (100), 79 (50), 77 (54), 67 (28), 55 (24), 41 (31).

12,15-dioxo-α-selinene (**3**): colorless oil; ^1^H NMR data consistent with literature [16]; ^13^C NMR (101 MHz, CDCl_3_) δ 194.78, 194.55, 154.82, 153.19, 142.04, 133.28, 43.47, 39.61, 37.02, 36.47, 32.14, 27.04, 26.34, 24.51, 15.76. EI-MS (70 eV) *m*/*z* (%): 232 (73, M^•+^), 214 (55), 203 (47), 199 (59), 185 (67), 171 (50), 107 (48), 105 (61), 95 (47), 93 (49), 91 (100), 81 (52), 79 (65), 77 (59), 67 (42), 55 (29), 41 (41).

14β,15β-dimethyl-7αH-eremophila-9,11-dien-8β-ol (**4**) EI-MS (70 eV) *m*/*z* (%): 220 (173, M^•+^), 189 (16), 152 (41), 149 (19), 137 (100), 123 (26), 122 (13), 121 (24), 109 (42), 108 (37), 107 (19), 105 (24), 96 (16), 91 (24), 81 (19), 55 (13), 41 (17). ^1^H and ^13^C NMR data: see Table 2.

### 3.6. Syntheses of Alcohol ***10*** and 7-epi-Neopetasane ***8***

Alcohol **10** was prepared from **9** [29] and was dehydrated according to published procedures [24]: 105 mg (0.44 mmoles, 1 eq.) of **10** was dissolved in dry pyridine (1.5 mL) and cooled in an ice bath. Thionyl chloride (62 mg, 0.52 mmoles, 1.17 eq.) was added to this mixture, and stirring was continued for 1.5 h at 0 °C. As the TLC control showed the disappearance of the starting materials, the reaction mixture was poured in a 10 mL saturated sodium hydrogen carbonate solution, and the mixture was extracted three times with MTBE. The combined organic phases were then washed successively with a 10% solution of acetic acid, water, saturated sodium hydrogen carbonate solution, and then brine. After drying on anhydrous magnesium sulfate, the solvent was evaporated, and the residue was purified by column chromatography on silica gel containing 10% silver nitrate to give pure dihydrokaranone **7** (13 mg) and 7-epi-neopetasane **8** (46 mg). 

Compound **10**: colorless oil; ^1^H NMR (400 MHz, CDCl_3_) δ 5.69 (s, 1H), 5.17 (s, 1H), 2.50 (dd, *J* = 13.0, 4.4 Hz, 1H), 2.39–2.24 (m, 2H), 2.01 (dd, *J* = 13.0, 4.4 Hz, 1H), 1.84–1.91 (m, 1H), 1.60–1.37 (m, 5H), 1.20 (2s, 6H), 1.12 (s, 3H), 0.91 (d, *J* = 6.4 Hz, 3H); ^13^C NMR (101 MHz, CDCl_3_) δ 203.44, 172.00, 124.64, 51.21, 43.64, 39.61, 38.65, 32.88, 30.35, 28.30, 26.99, 26.23, 24.59, 15.88, 15.17.

7-epi-neopetasane (**8**): colorless oil; ^1^H and ^13^C NMR data consistent with the literature for spectra measured in CDCl_3_ [21,32] or in C_6_D_6_ [33]. 

### 3.7. Reduction of 7-epi-Neopetasane ***8***

A total of 35 mg (0.16 mmoles, 1 eq.) of 7-epi-neopetasane (**8**) diluted in 0.5 mL absolute ethanol was added to a solution of sodium borohydride (19 mg, 0.49 mmoles, 3 eq.) in 1 mL absolute ethanol cooled in an ice bath, and the mixture was stirred at 0 °C for 10 min, and then at room temperature for 20 min. The reaction mixture was then cooled again to 0 °C, and ca. 100 μL of saturated sodium hydrogen carbonate solution was added. The excess of ethanol was then evaporated under vacuum on a rotatory evaporator, and 1 mL of MTBE and 1 mL of saturated sodium hydrogen carbonate solution were added to the residue. After shaking, the organic phase was collected, dried on magnesium sulfate, and evaporated to give a mixture of alcohols **5** and **11**, which was purified by column chromatography on silica gel to give a pure sample of **11** (4 mg) and a mixture of **11** and **5** (17 mg). (^13^C NMR data of **5** consistent with the literature [19].)

14β,15β-dimethyl-7βH-eremophila-9,11-dien-8β-ol (**11**): colorless oil; ^1^H NMR (400 MHz, CDCl_3_) δ 5.25 (pseudo t, *J* = 1.8 Hz, 1H), 4.84 (pseudo p, *J* = 1.6 Hz, 1H), 4.81 (d, *J* = 1.6 Hz, 1H), 4.01 (dt, *J* = 9.5, 2.0 Hz, 1H), 2.25–2.15 (overlapping peaks, 3H), 2.09–2.04 (multiplet, 1H), 1.71–1.68 (multiplet, 2H), 1.67 (t, *J* = 1.6 Hz, 3H), 1.58 (dd, *J* = 13.1, 2.6 Hz, 1H), 1.49–1.45 (multiplet, 1H), 1.36 (dd, *J* = 13.1, 3.0 Hz, 1H), 1.35–1.26 (multiplet, 1H), 0.94 (s, 3H), 0.76 (d, *J* = 6.7 Hz, 3H); ^13^C NMR (101 MHz, CDCl_3_) δ 146.69, 146.67, 122.23, 112.22, 68.97, 47.54, 44.14, 41.29, 38.92, 32.20, 30.93, 27.55, 19.41, 17.48, 15.25.

### 3.8. Oxidation of Alcohol ***4***

To a suspension of pyridinium chlorochromate (28 mg, 0.13 mmoles, 7 eq.) in 1 mL dichloromethane was added 4 mg (0.018 mmoles, 1 eq.) of alcohol **4** isolated from the SFE extract (see above). The suspension was stirred at room temperature, and the evolution of the reaction was followed by TLC. After 3h stirring, celite (10 mg) was added, and the reaction mixture was filtrated on a short pad of celite and silica gel, rinsed with MTBE, and the mixture was evaporated to furnish ca. 2 mg neopetasane **6** (^1^H NMR, ^13^C NMR, RI, and MS identical to a sample of pure neopetasane isolated from agarwood and consistent with the literature [21,30]).

### 3.9. Reduction of Neopetasane ***6***

The neopetasane (**6**) sample obtained above was reduced in the same conditions as **8** (see Section 3.7), and the resulting crude mixture was analyzed by ^1^H NMR and GC-MS. The characteristic signals of **4** could be detected in the NMR spectrum and in the GC-MS chromatogram.

## Figures and Tables

**Table 1:** Chemical composition of the SFE extract of Dongguan agarwood.

RI ^a^	RI_Litt_^b^	Compound	% ^c^	Identification ^d^
929	926	benzaldehyde	0.12	RI, MS
1012	1013	salicylaldehyde	0.03	RI, MS
1205	1199	4-phenyl-butan-2-one	0.20	RI, MS
1347	1347	vanillin	0.02	RI, MS
1457	1474	4,5-diepi-aristolochene	0.09	RI, MS
1595	1607	10-epi-γ-eudesmol	0.14	RI, MS
1608	1611	γ-eudesmol	0.11	RI, MS
1612	1628	agarospirol	0.43	RI, MS
1615	1631	hinesol	0.27	RI, MS
1620		jinkoh eremol	0.34	RI, MS
1624	1628	β-eudesmol	0.98	RI, MS
1628	1636	kusunol (valerianol)	1.24	RI, MS
1640		14β,15β-dimethyl-7αH-eremophila-9,11-dien-8β-ol **4**	0.67	RI, MS, NMR
1655		isobaimuxinal	0.55	RI, MS
1711		neopetasane **6**	0.72	RI, MS, NMR
1716		baimuxinol	0.02	RI, MS
1738	1738	cryptomeridiol	1.20	RI, MS
1763		dihydrokaranone **7**	0.80	RI, MS, NMR
1791	1786	oxo-agarospirol (baimuxinal) **1**	1.90	RI, MS
1835		dihydrocolumellarin	0.90	RI, MS
1896		12,15-dioxo-α-selinene **3**	1.58	RI, MS, NMR
1924		12,15-dioxo-selinan-4,11-diene **2**	2.27	RI, MS, NMR
1964		1,5-diphenyl-3-pentanone	0.08	RI, MS
2106		1,5-diphenyl-1(*E*)-penten-3-one	0.35	RI, MS
2266	2296	2-(2-phenylethyl)chromone	11.01	RI, MS
2464		5,8-dihydroxy-2-(2-phenylethyl)chromone ^e^	0.08	MS
2493		2-(methoxyphenylethyl)chromone ^f^	0.15	MS
2524		methoxy-2-(2-phenylethyl)chromone ^f^	4.02	MS
2533		2-(methoxyphenylethyl)chromone ^f^	3.62	MS
2562		methoxy-2-(2-phenylethyl)chromone ^f^	0.10	MS
2772		6-methoxy-2-(4-methoxyphenethyl)chromone ^e^	0.13	MS
2782		6,7-dimethoxy-2-(2-phenylethyl)chromone ^e^	1.69	MS
2808		squalene	0.12	MS
3018		dimethoxy-2-(methoxyphenethyl)chromone ^f^	0.11	MS

^a^ RI = linear retention indices determined in relation to a homologous series of n-alkanes (C_8_–C_40_) on an RTX-1 column. ^b^ RI_Litt_ = linear retention index from the literature (when available), not indicated when the retention index was already listed in the homemade retention index library built with authentic reference compounds (see ^d^). ^c^ Mass percentage determined by GC-FID using predicted response factors [11], with tetradecane as an internal reference. ^d^ Identification method: MS = constituent identified by comparison of its mass spectrum with that listed in the literature or in a homemade mass spectral library built with authentic reference compounds; RI = constituent identified by comparison of its retention index with that listed in the literature or in a homemade retention index library built with authentic reference compounds; NMR = the structure of the identified constituent was confirmed by 1D and 2D NMR analysis in the frame of the present study. ^e^ Substitution pattern deduced from [12]. ^f^ Accurate position of the methoxy substituents on the chromone or the phenylethyl moiety not determined.

**Table 2:** ^1^H, ^13^C, ^1^H-^1^H COSY, and ^1^H-^13^C HMBC NMR spectral data for compound **4** in CDCl_3_ (carbon atom numbers given in Figure 2).

No ^a^	NMR Data			
	^13^C	^1^H	COSY	HMBC
1a	32.23	2.01–1.95 (multiplet, 1H)	H-2	C-2, C-3, C-9, C-10
1b		2.30–2.21 (multiplet, 1H)	H-2	
2a	29.31	1.36–1.24 (multiplet, 1H)	H-1	C-1, C-10
2b		1.87–1.80 (multiplet, 1H)	H-1	
3a	31.07	1.60–1.46 (overlapping peaks, 2H)	/ ^b^	/ ^b^
3b		1.73–1.66 (overlapping peaks, 2H)		
4	37.61	1.64–1.58 (multiplet, 1H)	H-15	C-5, C-15
5	39.66	/	/	/
6a	31.27	1.60–1.46 (overlapping peaks, 2H)	/ ^b^	/ ^b^
6b		1.73–1.66 (overlapping peaks, 2H)		
7	41.95	2.21–2.11 (multiplet, 1H)	H-8	C-9, C-10
8	64.20	4.00 (ddd, *J* = 5.9, 3.1, 1.3 Hz, 1H)	H-7, H-9	
9	119.09	5.54 (dd, *J* = 6.0, 1.3 Hz, 1H)	H-8	C-1, C-5, C-7, C-8
10	152.34	/	/	/
11	146.45	/	/	/
12a	111.60	4.94–4.91 (multiplet, 1H)	H-13	C-7, C-13
12b		4.78–4.76 (multiplet, 1H)	H-13	
13	22.78	1.76 (broad s, 3H)	H-12	C-7, C-11, C-12
14	20.82	0.92 (s, 3H)	/	C-4–C-6
15	15.98	0.76 (d, *J* = 6.7 Hz, 3H)	H-4	C-3–C-5

^a^ Number of carbon atoms (see Figure 2). ^b^ Determination of the interactions is not confirmed due to the peaks overlapping.

## Data Availability

Data are contained within the article and Supplementary Materials, and can be provided by the corresponding author on request.

## Supplementary Materials

The following supporting information can be downloaded at: https://www.mdpi.com/article/10.3390/molecules29102297/s1, Figures S1–S14: ^1^H and ^13^C NMR charts for compounds **2**–**4**, **6**, **8**, **10**–**11**.
