# Supplementary material for: Chemical Investigation on the Volatile Part of the CO2 Supercritical Fluid Extract of Infected Aquilaria sinensis (Chinese Agarwood)
Source: Molecules. 2024 May 14;29(10):2297. doi: 10.3390/molecules29102297 (PMC11124402; doi:10.3390/molecules29102297)
Supplement: Supplementary file 1 [file molecules-29-02297-s001.zip › molecules-2979106-supplementary.pdf]

Supplementary material

for

Chemical investigation on the volatile part of the CO_2 super-critical fluid extract of infected *Aquilaria sinensis* (Chinese agarwood)

Marko Z. Mladenović [1], Ou Huang [2], Bo Wang [3], Alexandre Ginestet [4], Didier Desbiaux [4], and Nicolas Baldovini [5,*]

[1] Faculty of Sciences and Mathematics, University of Niš, Višegradska 33, 18000 Niš, Serbia; markohem87@gmail.com
[2] Guangdong Shangzhengtang Group Co. Ltd, 6 Qian Wu street, Dongcheng road, 523129 Dongguan, China; sztchina@163.com
[3] School of Life and Health Technology, Dongguan University of Technology, 523808 Dongguan, China; bwang@dgut.edu.cn
[4] MAG Industry Holdings Ltd., 26 Queensland, Nancheng Century City, 523617 Dongguan, China; didier.desbiaux@gmail.com (D.D.); alexandre@mag-vision.fr (A.G.)
[5] Institut de Chimie de Nice. UMR 7272. Université Côte d'Azur. Parc Valrose 06108 Nice. France; Nicolas.BALDOVINI@univ-cotedazur.fr

* Correspondence: Nicolas.BALDOVINI@univ-cotedazur.fr; Tel.: +334 92076133

Content:

Figure S1. ^{1}H NMR spectrum of (**2**) recorded in $CDCl_3$
Figure S2. ^{13}C NMR spectrum of (**2**) recorded in $CDCl_3$
Figure S3. ^{1}H NMR spectrum of (**3**) recorded in $CDCl_3$
Figure S4. ^{13}C NMR spectrum of (**3**) recorded in $CDCl_3$
Figure S5. ^{1}H NMR spectrum of (**4**) recorded in $CDCl_3$
Figure S6. ^{13}C NMR spectrum of (**4**) recorded in $CDCl_3$
Figure S7. ^{1}H NMR spectrum of (**6**) recorded in $CDCl_3$
Figure S8. ^{13}C NMR spectrum of (**6**) recorded in $CDCl_3$
Figure S9. ^{1}H NMR spectrum of (**8**) recorded in $CDCl_3$
Figure S10. ^{13}C NMR spectrum of (**8**) recorded in $CDCl_3$
Figure S11. ^{1}H NMR spectrum of (**10**) recorded in $CDCl_3$
Figure S12. ^{13}C NMR spectrum of (**10**) recorded in $CDCl_3$
Figure S13. ^{1}H NMR spectrum of (**11**) recorded in $CDCl_3$
Figure S14. ^{13}C NMR spectrum of (**11**) recorded in $CDCl_3$

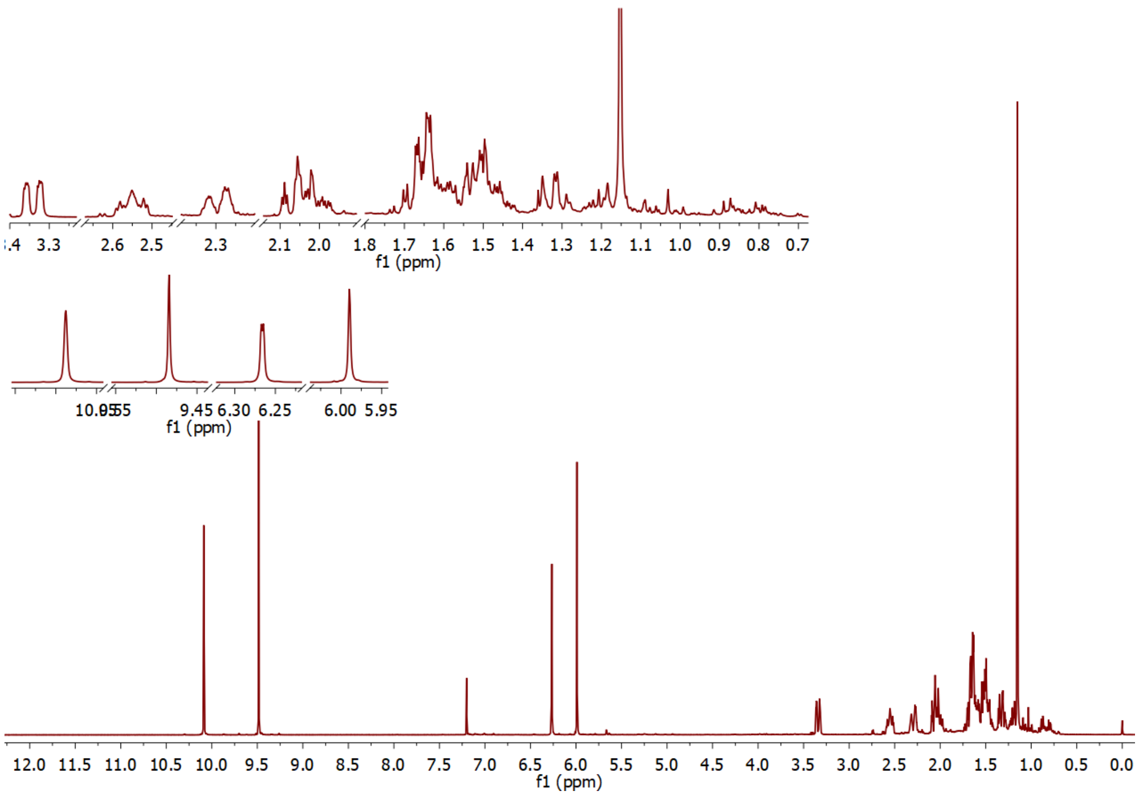

Figure S1. ^{1}H NMR spectrum of (**2**) recorded in $CDCl_3$

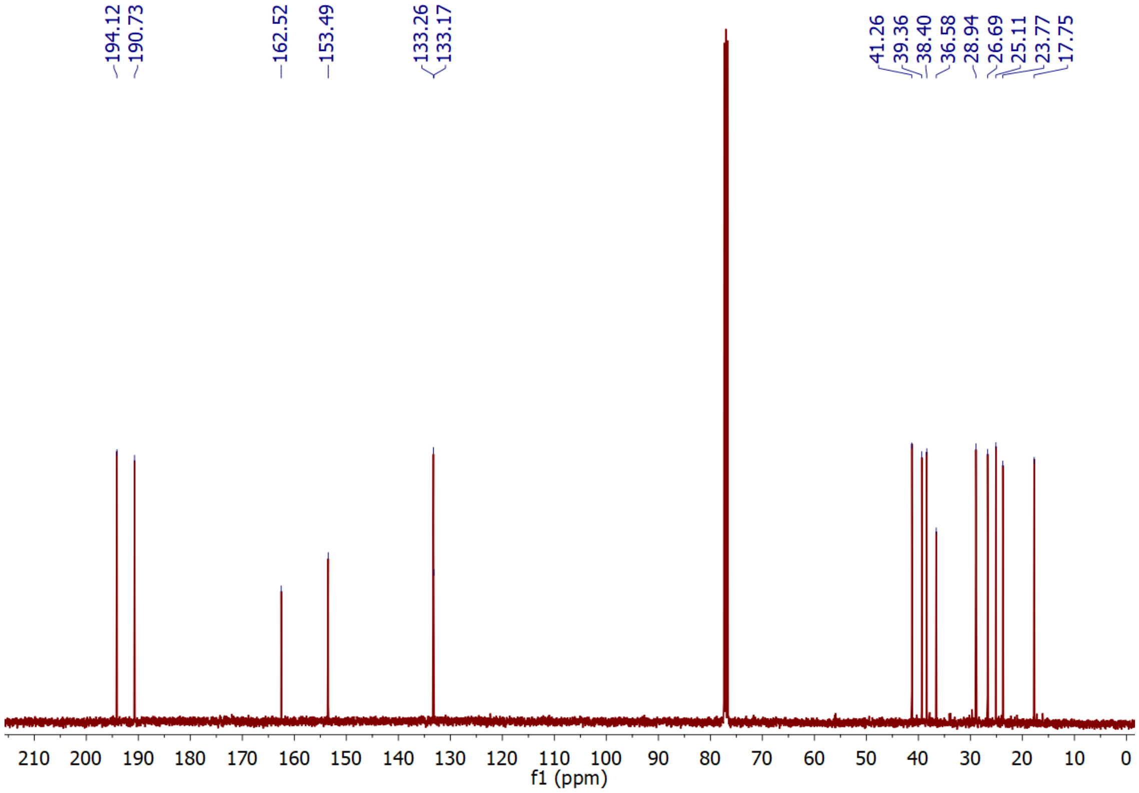

Figure S2. ^{13}C NMR spectrum of (**2**) recorded in $CDCl_3$

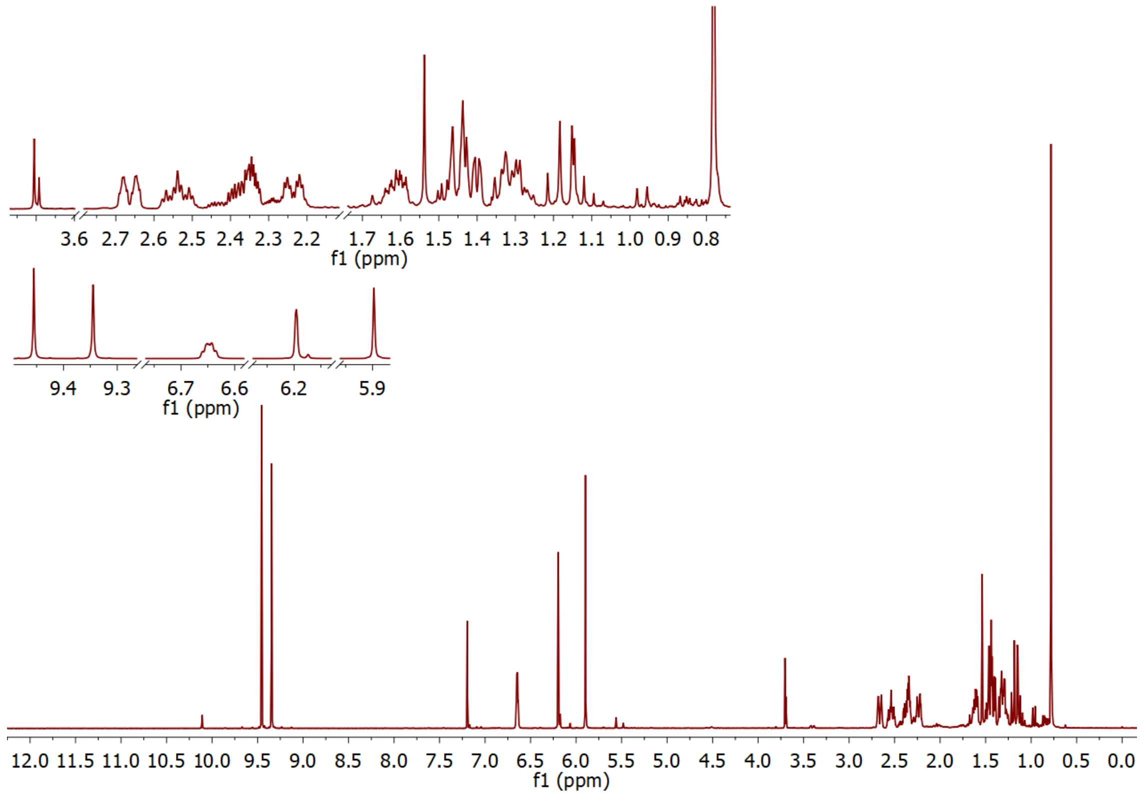

Figure S3. ^{1}H NMR spectrum of (**3**) recorded in $CDCl_3$

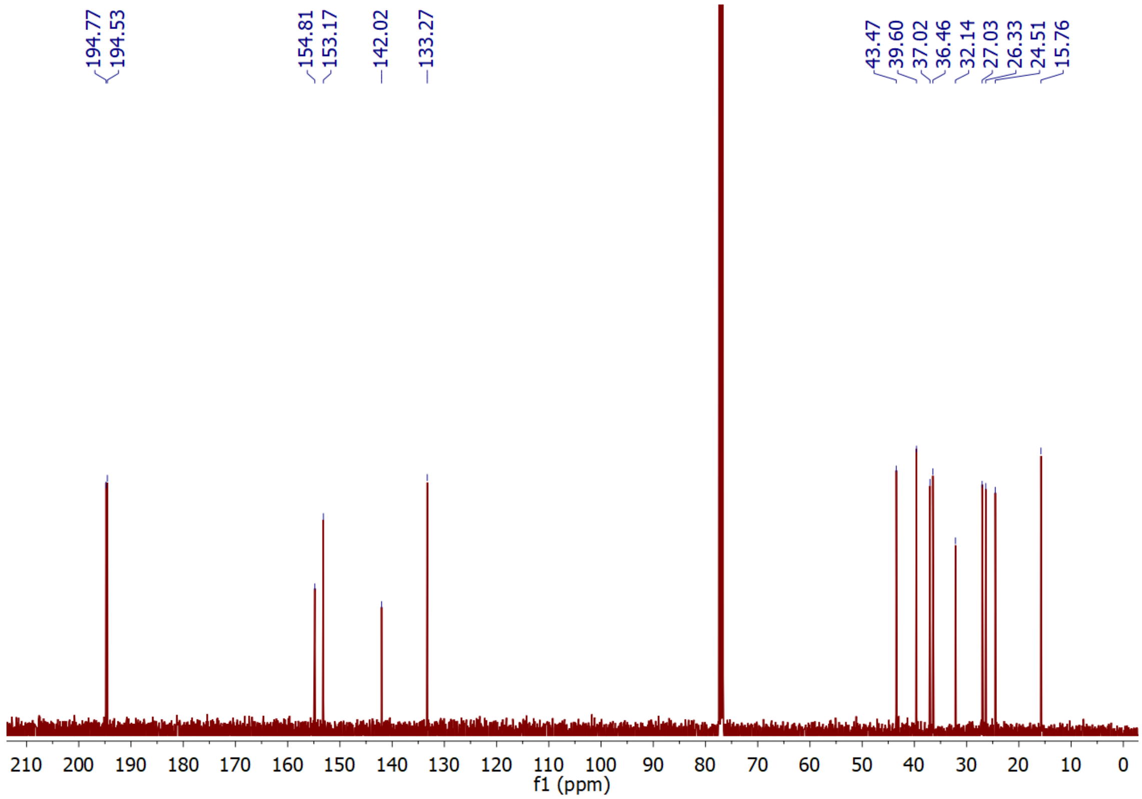

Figure S4. ^{13}C NMR spectrum of (**3**) recorded in $CDCl_3$

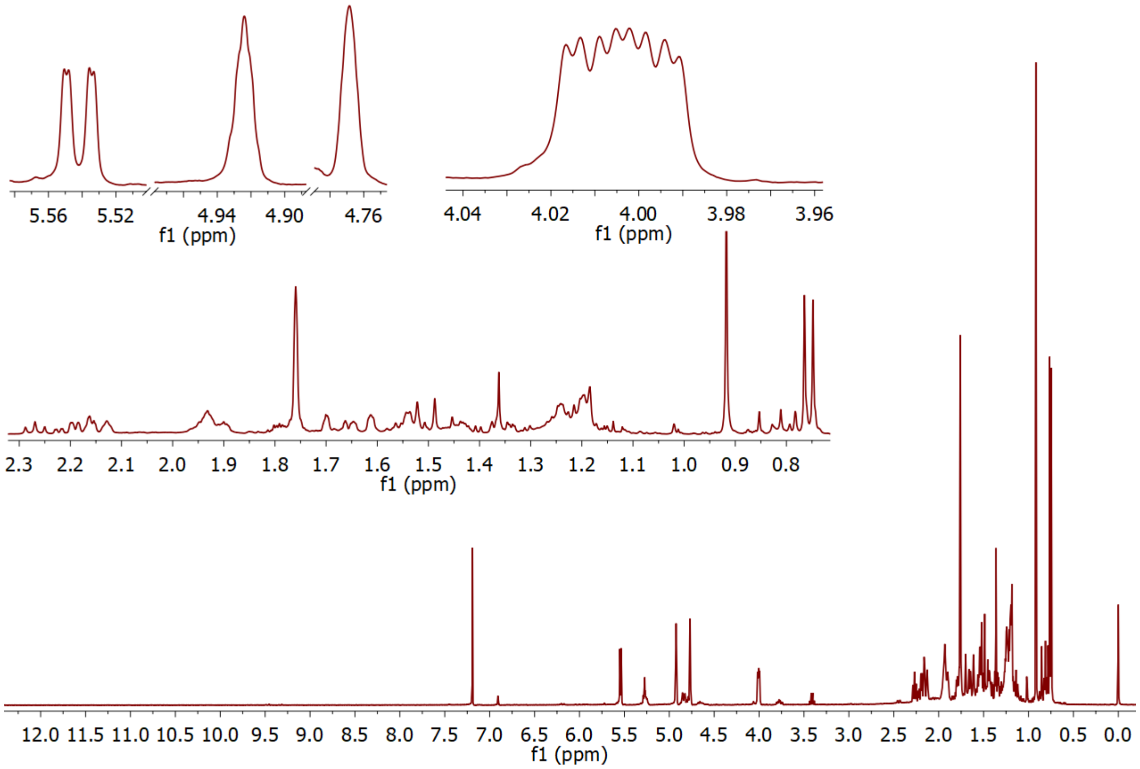

Figure S5. ^{1}H NMR spectrum of (**4**) recorded in $CDCl_3$

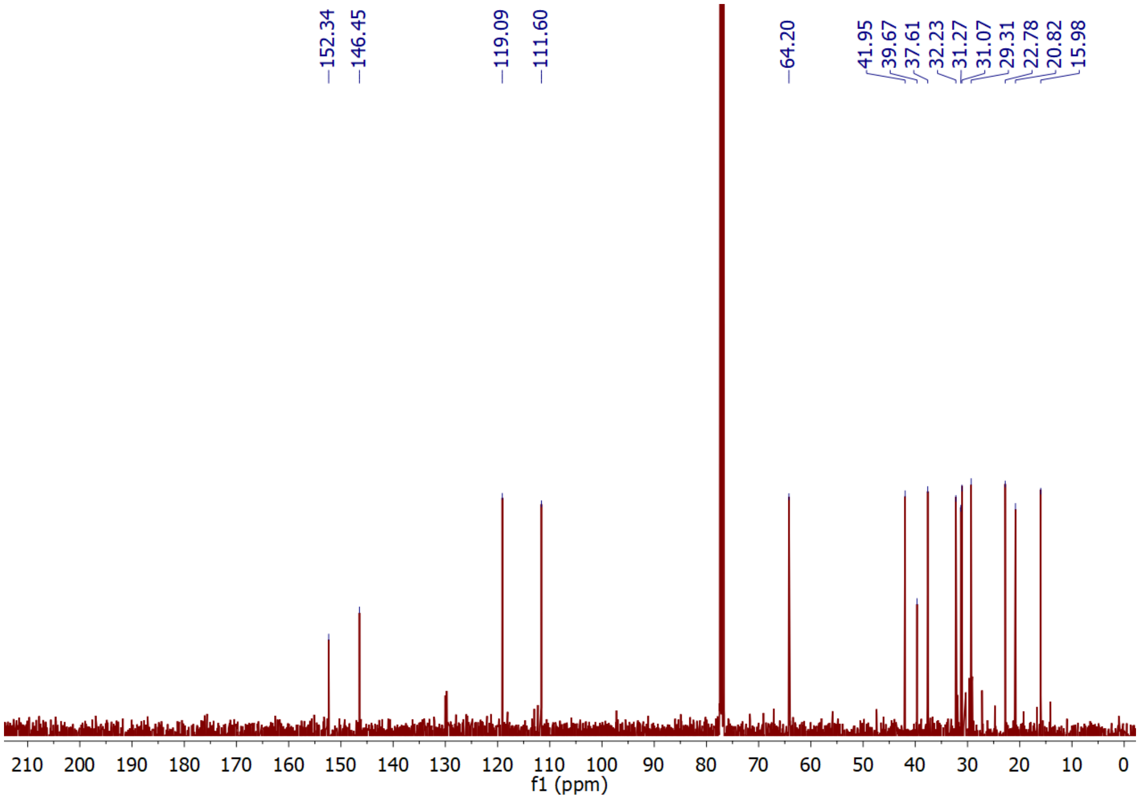

Figure S6. ^{13}C NMR spectrum of (**4**) recorded in $CDCl_3$

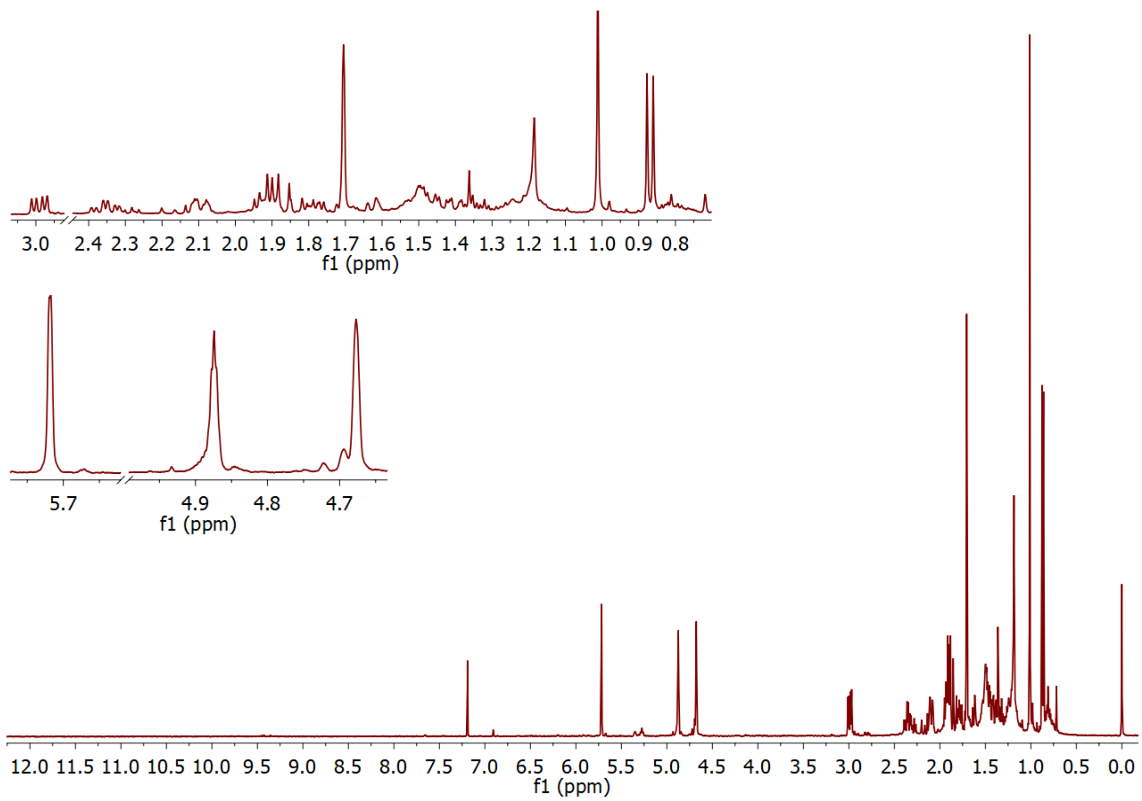

Figure S7. ^{1}H NMR spectrum of (**6**) recorded in $CDCl_3$

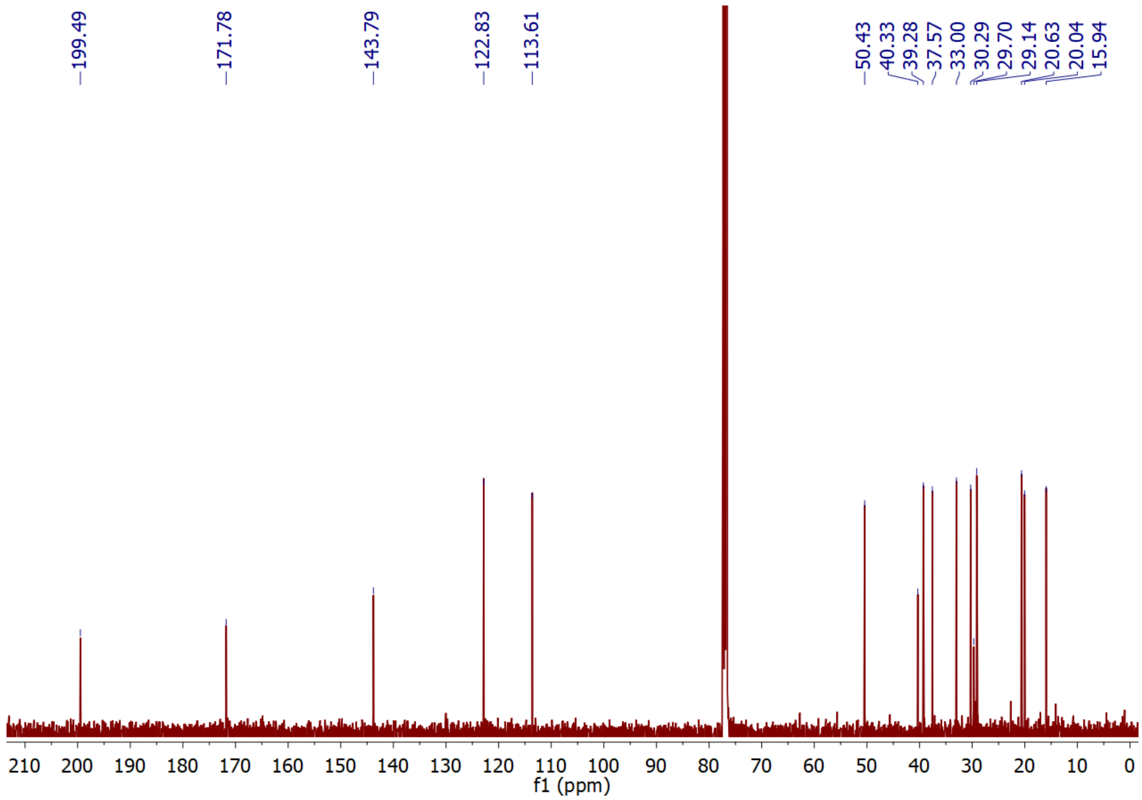

Figure S8. ^{13}C NMR spectrum of (**6**) recorded in $CDCl_3$

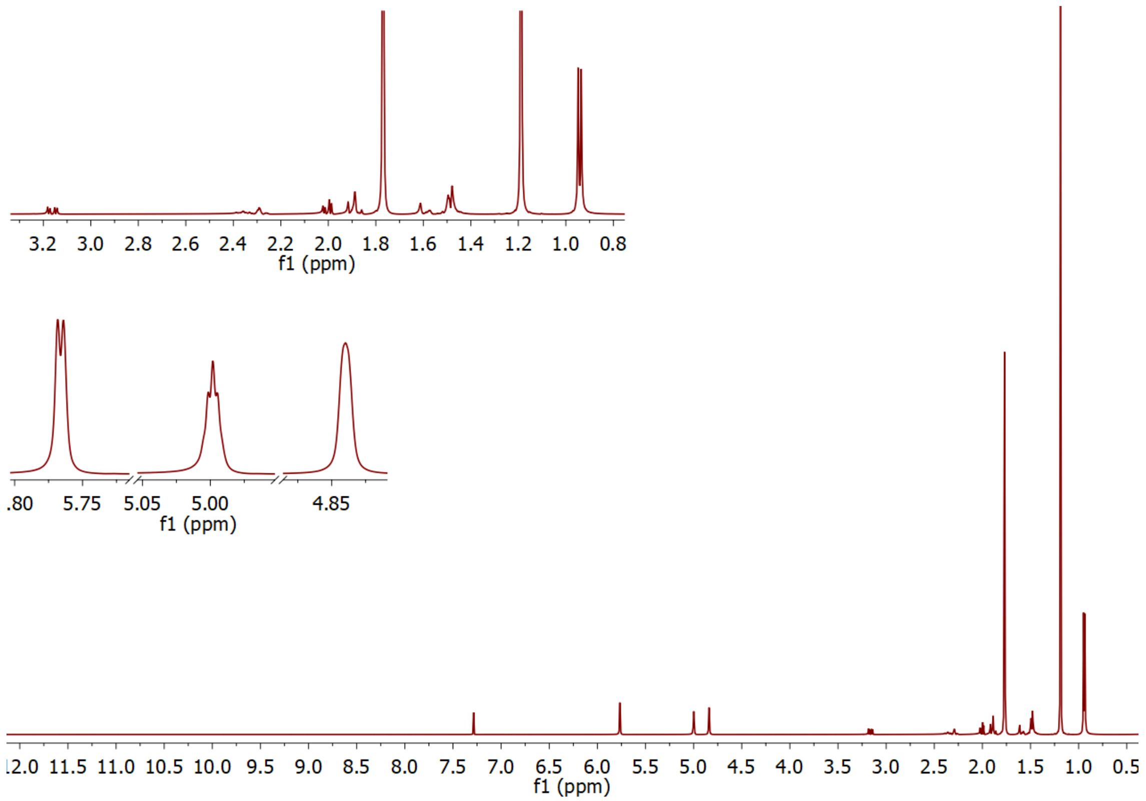

Figure S9. ^{1}H NMR spectrum of (**8**) recorded in $CDCl_3$

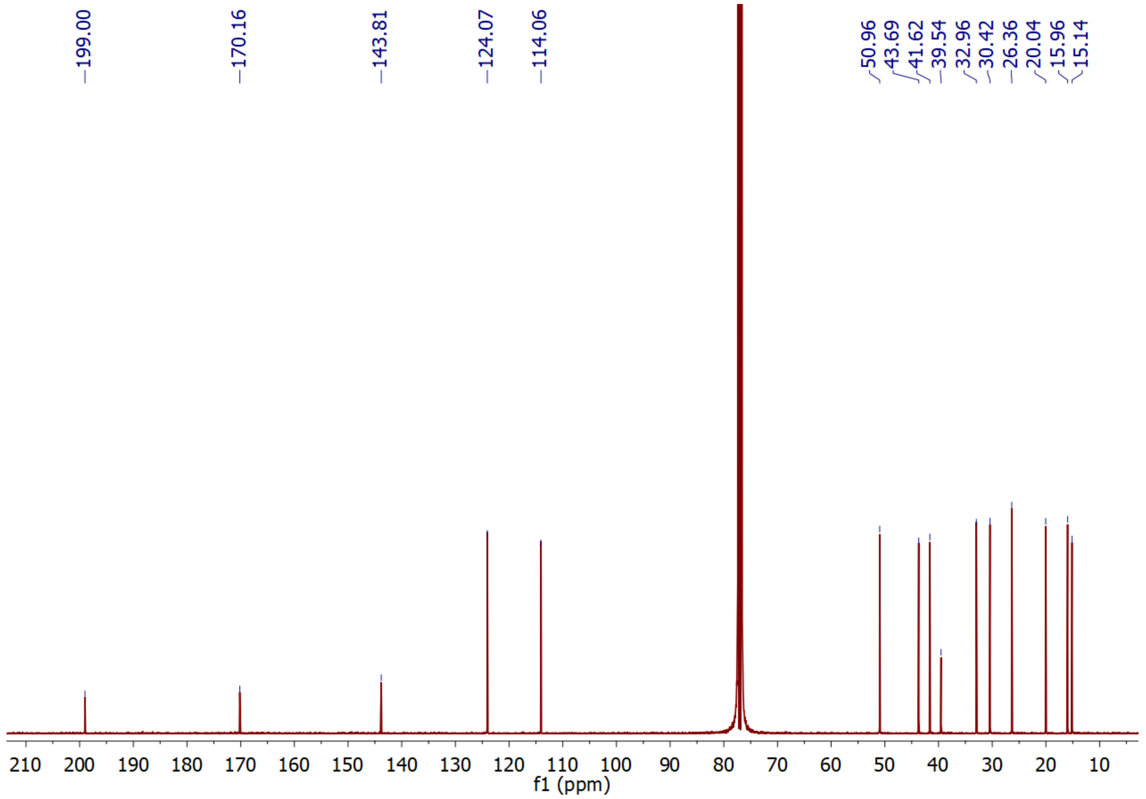

Figure S10. ^{13}C NMR spectrum of (**8**) recorded in $CDCl_3$

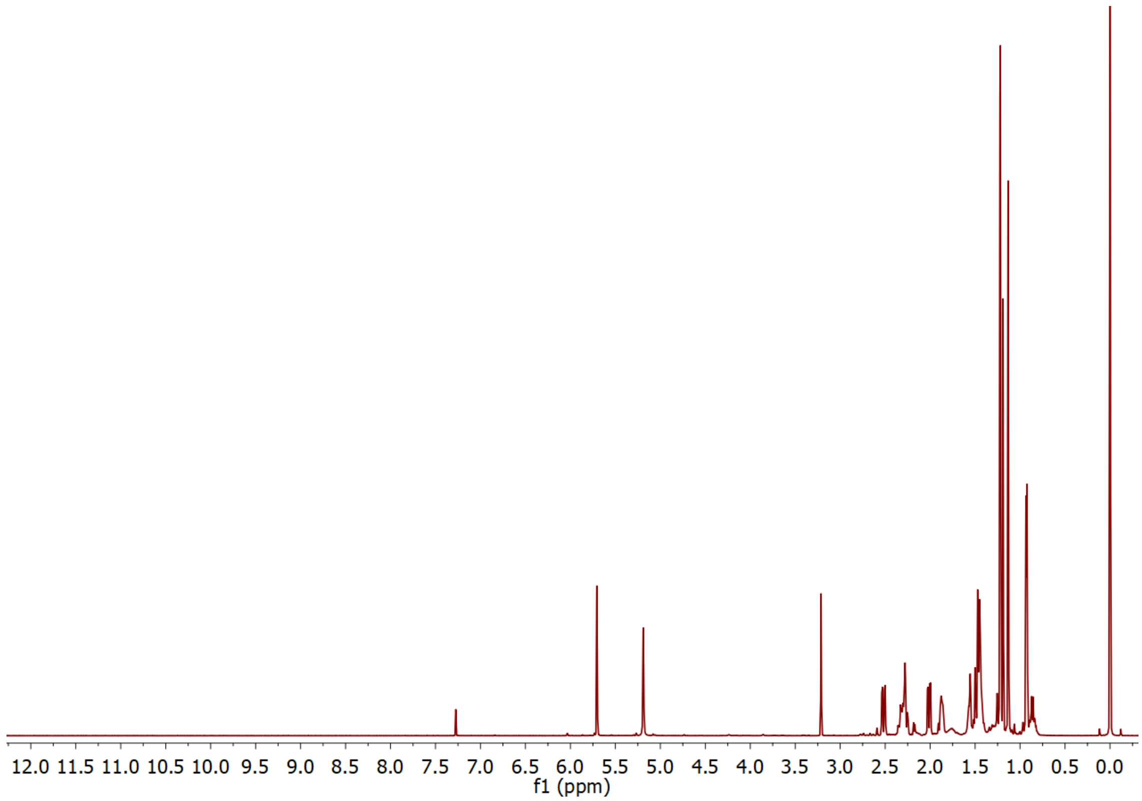

Figure S11. ^{1}H NMR spectrum of (**10**) recorded in $CDCl_3$

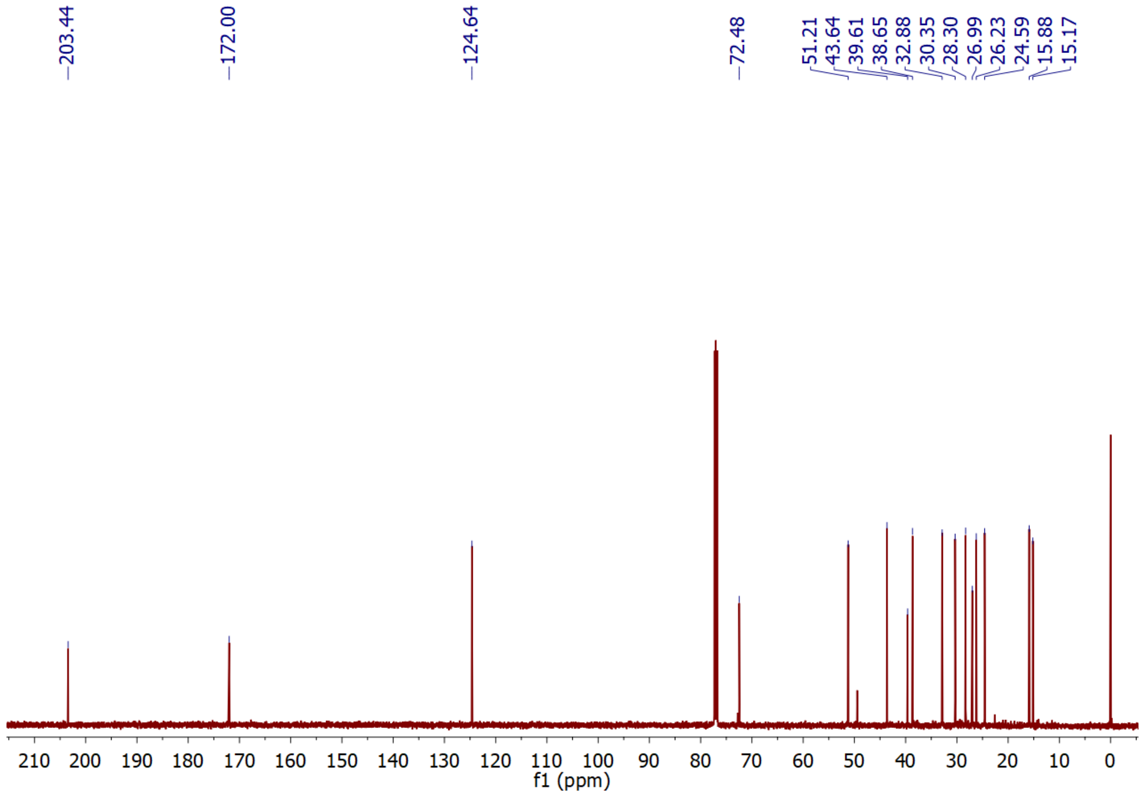

Figure S12. ^{13}C NMR spectrum of (**10**) recorded in $CDCl_3$

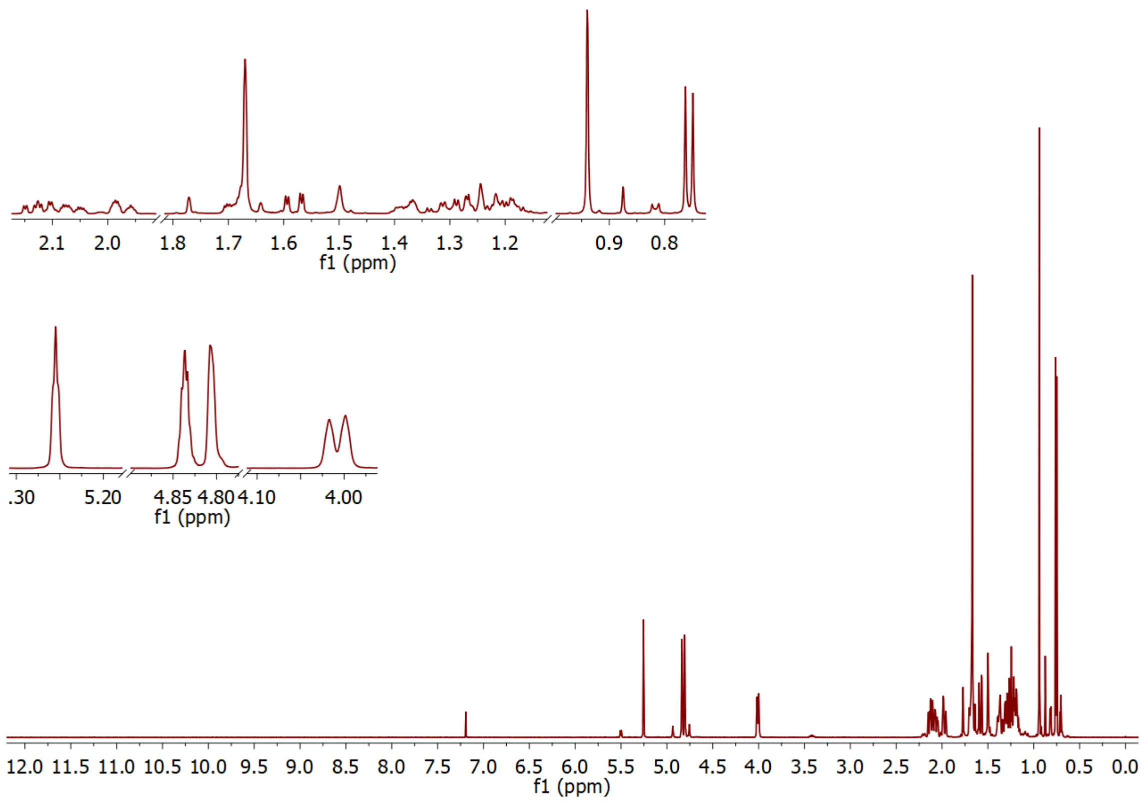

Figure S13. ^{1}H NMR spectrum of (**11**) recorded in $CDCl_3$

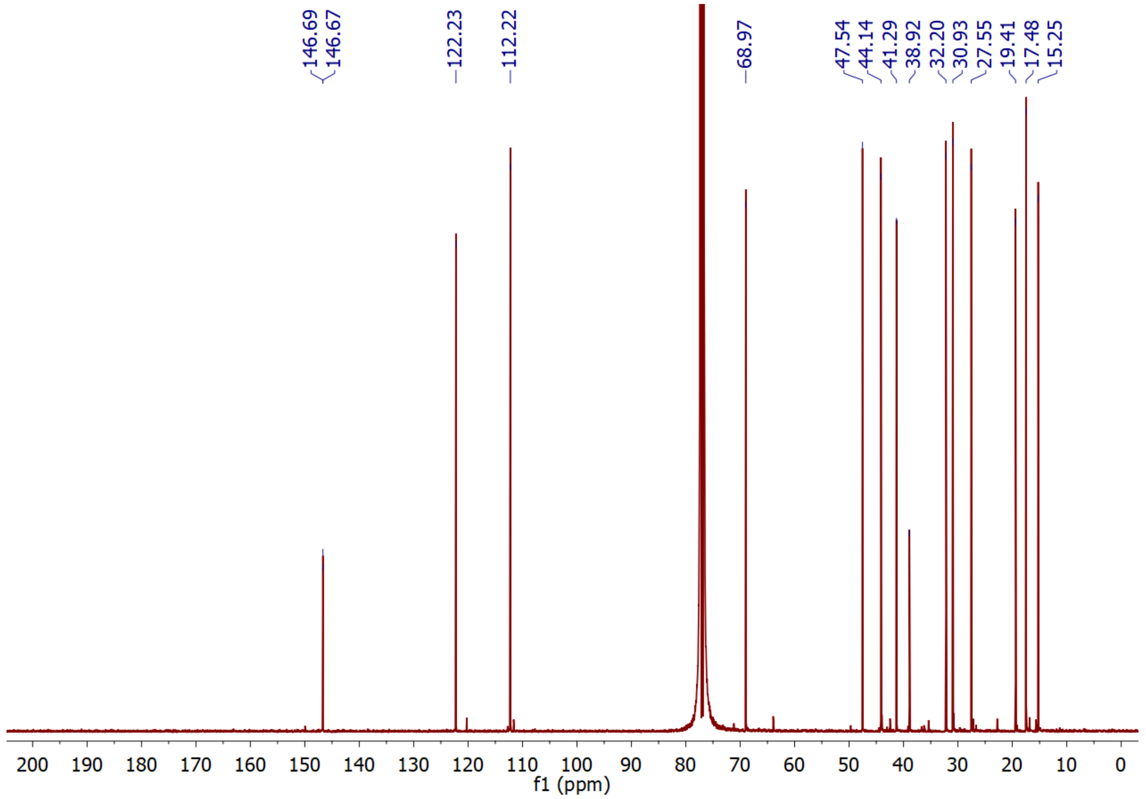

Figure S14. ^{13}C NMR spectrum of (**11**) recorded in $CDCl_3$
